# Outcomes of nurse practitioner‐led care in patients with cardiovascular disease: A systematic review and meta‐analysis

**DOI:** 10.1111/jan.14229

**Published:** 2019-10-24

**Authors:** Marcie J. Smigorowsky, Meghan Sebastianski, Michael Sean McMurtry, Ross T. Tsuyuki, Colleen M. Norris

**Affiliations:** ^1^ Mazankowski Alberta Heart Institute Edmonton Alberta Canada; ^2^ Department of Pediatrics Alberta Strategy for Patient‐Oriented Research SUPPORT Unit, Knowledge Translation Platform University of Alberta Edmonton Alberta Canada; ^3^ Division of Cardiology Faculty of Medicine and Dentistry University of Alberta Edmonton Alberta Canada; ^4^ Faculty of Nursing University of Alberta Edmonton Alberta Canada

**Keywords:** cardiovascular care, clinical intervention, meta‐analysis, nurse, nurse practitioner, outcomes of care, randomized control trial, systematic review

## Abstract

**Aim:**

To assess randomized controlled trials evaluating the impact of nurse practitioner‐led cardiovascular care.

**Background:**

Systematic review of nurse practitioner–led care in patients with cardiovascular disease has not been completed.

**Design:**

Systematic review and meta‐analysis.

**Data sources:**

The Cochrane Central Register of Controlled Trials (CENTRAL), Medline, Embase, CINAHL, Web of Science, Scopus and ProQuest were systematically searched for studies published between January 2007 ‐ June 2017.

**Review Methods:**

Cochrane methodology was used for risk of bias, data extraction and meta‐analysis. The quality of evidence was assessed using Grading of Recommendations Assessment, Development and Evaluation approach.

**Results:**

Out of 605 articles, five articles met the inclusion criteria. There was no statistical difference between nurse practitioner‐led care and usual care for 30‐day readmissions, health‐related quality of life and length of stay. A 12% reduction in Framingham risk score was identified.

**Conclusion:**

There are a few randomized control trials assessing nurse practitioner‐led cardiovascular care.

**Impact:**

Low to moderate quality evidence was identified with no statistically significant associated outcomes of care. Nurse practitioner roles need to be supported to conduct and publish high‐quality research.

## Abstract (zh)

**目的:**

在于评估随机对照试验,从而评估执业护士主导型护理心血管护理的影响。

**背景:**

尚未完成对心血管疾病患者进行执业护士主导型护理的系统综述。

**设计:**

系统综述和荟萃分析。

**资料来源:**

在 Cochrane Central Register of Controlled Trials (CENTRAL)、Medline、Embase、CINAHL、Web of Science、Scopus和ProQuest上系统地搜索了2007年1月至2017年6月间发表的研究。

**综述方法:**

Cochrane方法学用于偏倚风险、数据提取和荟萃分析。证据质量的评估采用了GRADE(Grading of Recommendations Assessment, Development and Evaluation)法。

**结果:**

在605篇文章中,有5篇符合纳入标准。执业护士主导型护理和30天再度入院的常规护理、健康相关生活质量和住院天数之间没有统计学差异。确定Framingham 风险评分降低了12%。

**结论:**

有一些随机对照试验评估了执业护士主导型心血管护理。

**影响:**

确定了低等至中等质量的证据,没有统计学意义的相关护理结果。需要支持执业护士的角色,从而开展和发布高质量研究。

## 1 INTRODUCTION

Healthcare reform is occurring internationally, rooted in important issues such as reducing healthcare costs, wait times for appointments and procedures, and improving quality of care and patient safety (Gibbons et al., 2008). While healthcare reform has been occurring for the last twenty years, there are two parallel issues which contribute to barriers for swift and successful change. Globally, most countries have an ageing population and burgeoning growth of people living with chronic diseases (Advisory Panel on Healthcare innovation, 2015). Individually and together, these two health issues continue to increase use further taxing increasingly limited healthcare resources worldwide.

Sky rocketing healthcare costs are also creating ongoing challenges to healthcare sustainability (Simms, 2010). Healthcare leaders and providers are therefore looking for new innovative models of care to give safe and affordable patient care. A question is often asked: why use one healthcare provider role over another? With limited healthcare dollars, leaders have to make justifications to determine which model of care to use. Healthcare leaders are encouraged to use healthcare data and outcomes to inform difficult decisions; such as the use of healthcare providers (Ellis, 2015). In Canada (Advisory Panel on Healthcare innovation, 2015) Australia (Boase, 2019), the United Kingdom (Reynolds & Mortimore, 2018) and the United States (Institute for Healthcare Improvement, 2019) there is growing support for healthcare providers working to their full scope of practice and therefore it is essential to clearly outline the benefits of specific roles.

Internationally nurse practitioners (NP) are graduate‐level prepared registered nurses (in most countries), whose scope of practice includes health maintenance and promotion from diagnosis, treatment, to follow‐up of patients with acute and chronic conditions in both the inpatient and outpatient setting (Canadian Nurses Association, 2002; College & Assocation of Registered Nurses of Alberta, 2017). Nurse practitioners are independent practitioners but often work in collaborative healthcare teams. NPs are unique because they use select skills from medicine and advanced nursing skills that may result in greater benefits to patients and the healthcare system. Benefits may include: decreased costs, increased patient engagement with their care and improved quality of life (Shuler & Davis, 1993).

Currently NP‐led cardiovascular (CV) care and the associated outcomes of care have not been broadly evaluated. Randomized controlled trials (RCT) are the gold standard to evaluate treatment efficacy (Meldrum, 2000). Therefore, the purpose of this study was to conduct a systematic review (SR) and meta‐analysis of RCTs assessing NP‐led CV care and associated outcomes of care.

### 1.1 Background

Nurse Practitioners are able to diagnose, prescribe and independently order treatments (College & Assocation of Registered Nurses of Alberta, 2017). The NP role was implemented in the late 1960s in the United States (American Association of Nurse Practitioners, 2017) and in Canada during the late 1970s (Spitzer et al., 1974). The focus of the role initially was in primary care and pediatrics (Hayes, 1985; Spitzer et al., 1974). In the late 1980’s the acute NP role was introduced which led to NPs practicing in the hospital setting (Kilpatrick et al., 2010) focusing typically on specialty areas of care such as CV care (Broers et al., 2009; Stables et al., 2004; Tranmer & Parry, 2004). In many tertiary centres, NPs give care in CV settings in cardiology and CV surgery. Cardiovascular NP’s specialize in the diagnosis and treatment of heart disease/abnormalities and postoperative heart surgical care in intensive care, ward settings and ambulatory outpatient clinics.

Studies have reported the use of the NP role results in outcomes associated with healthcare reforms such as increased patient satisfaction, improved patient outcomes, decreased length of stay and improved health‐related quality of life (HRQOL) (Delamaire & Lafortune, 2010; Donald et al., 2014; Kilpatrick et al., 2010; Laurant et al., 2009; Litaker et al., 2003). A systematic review on the safety and effectiveness of NP‐led care in primary care included seven RCTs, two economic analyses and one follow‐up study. A quality assessment does not appear to have been completed however, they identified NP‐led care was associated with equal or slightly better outcomes compared with physician‐led care for physiologic measures (e.g. improved BP and cholesterol control) patient satisfaction and cost. NP‐led care was also associated with slightly longer consultations than physician‐led care (Swan, Ferguson, Chang, Larson, & Smaldone, 2015). The final conclusion was that NP‐led care was effective and safe. A recent Cochrane Library SR was completed evaluating NPs (and other nursing roles) as substitutes for physicians in primary care (Laurant et al., 2018). Eighteen RCTs were included. Results aligned with the findings from the Swan et al. SR and suggested that there are similar or better patient outcomes associated with NP‐led care compared with physician‐led care. NP‐led care is also associated with increased patient satisfaction, longer consultations and possibly higher return visits, but there is no difference for hospital admission, emergency room visits, number of prescriptions filled and number of tests ordered. While the evidence for primary care NP‐led care is growing, there is a gap in the evidence specifically in understanding the specific areas in CV care where NPs currently give care to patients and the associated outcomes of the roles.

We completed an a priori comprehensive review (literature searched from years January 1980 ‐ February 2017) to try to establish the typical CV NP‐led care outcomes. The initial search identified 2040 studies. After title review, the search identified 170 studies (all types) identifying different models of care with significant methodological issues. With the current interest in using the NP role, we felt that a systematic review of RCTs comparing NP‐led care versus other models of care (typically physician‐led/usual care) in any CV setting was required to identify CV NP‐led care as a model of care and possible associated outcomes.

Using an NP in CV care, may be a well‐founded option to meet the increasing demands that the expanding prevalence of CV disease is placing on the healthcare system. Acquiring a better understanding of the types of roles and potential clinical outcomes of care will be helpful to healthcare leaders to assist with further development and use of NP‐led CV care.

## 2 THE REVIEW

### 2.1 Aim

This systematic review and meta‐analysis aimed to appraise the existing evidence related to the effectiveness of cardiovascular nurse practitioner‐led care (as a model of care) on the outcomes of care for adult patients. The research question is: what are the outcomes of care associated with cardiovascular nurse practitioner‐led care?

### 2.2 Design

This research was conducted by completing a SR of RCTs reporting NPs providing care in CV patient care settings and examining the impact of clinical outcomes of care associated with NP‐led care using the guidelines of the Cochrane Collaboration (Higgins et al., 2019) and reported using Preferred Reporting Items for Systematic Reviews and Meta‐Analyses (PRISMA) statement (Moher et al., 2015).

### 2.3 Search methods

All published and unpublished RCTs related to CV NP‐led care and associated outcomes of care between January 2007 and July 2017 (years) were identified in the following databases: CINAHL, EMBASE, Medline, ProQuest Dissertations & Thesis Global, the Cochrane Central Register of Controlled Trials (CENTRAL), Scopus and Web of Science Core Collection. We consulted with a librarian familiar with nursing and medical research when we developed and conducted the search. Search results were limited to English and relevant references in articles reviewed were also assessed. The full search strategy is attached as Appendix S1A. Limiting the time frame to the last 10 years was important because healthcare delivery has changed over this time period as has the increased implementation and acceptance of the NP role (Dill, Pankow, Erikson, & Shipman, 2013; Peterson, Phillips, Puffer, Bazemore, & Petterson, 2013; The NP Integration Research (NPIR) Team, 2015). The following combinations of MeSH (Medical Subject Heading) terms or keywords were used: cardiovascular disease, atrial fibrillation, nurse practitioner, randomized controlled trial, cardiology, cardiac surgery, coronary artery disease, high cholesterol and hypertension. Stroke was considered initially however, we felt it was a separate focus of care and therefore excluded in the title review. Duplicate records and trials were excluded by screening the titles and abstracts. The remaining articles were reviewed to determine if they met inclusion criteria.

#### 2.3.1 Participants

Studies were included  with the following inclusion criteria: greater than 18 years of age, diagnosed with cardiovascular disease (e.g. coronary artery disease, cardiac risk reduction, cardiac arrhythmias, congenital heart disease, cardiomyopathy, heart failure, cardiac surgery or interventional cardiology), randomly allocated to either CV NP‐led care or another CV healthcare provider.

#### 2.3.2 Interventions

The NP had to be the lead care provider for patients in the intervention group either as an: independent practitioner or a member of an interprofessional healthcare team. The NP may have completed assessments, diagnosed new findings, ordered and monitored medications/diagnostic testing and completed interventions. The NP could also have consulted other healthcare professionals to give specialty services. (e.g. a physiotherapist or a physician).

#### 2.3.3 Comparison

The comparison group received care led by another CV healthcare provider (typically physician‐led care but could be a physician assistant or other model of care).

#### 2.3.4 Outcomes

Outcomes of care in this review had to be associated with NP‐led care specific to the setting and focus of the assessed research study. Identified outcomes depended on what were reported. We hypothesized identified outcomes may include: change in symptoms (e.g. shortness of breath, angina, palpitations), change in monitored risk factor reduction variables (e.g. blood pressure, cholesterol panel values), healthcare system quality improvement (e.g. wait times, length of stay) and patient reported (e.g. quality of life, patient satisfaction).

#### 2.3.5 Types of studies

This review included only RCTs because we were looking at role effectiveness and felt that it was important to find evidence at the highest level to understand outcomes associated with NP‐led care compared with another healthcare provider. Randomized controlled trials are also the most rigorous way to determine if a cause‐effect relationship exists between the intervention and outcome (s) (Nelson, 2011; Sibbald & Roland, 1998).

### 2.4 Search outcome

**Figure 1:**
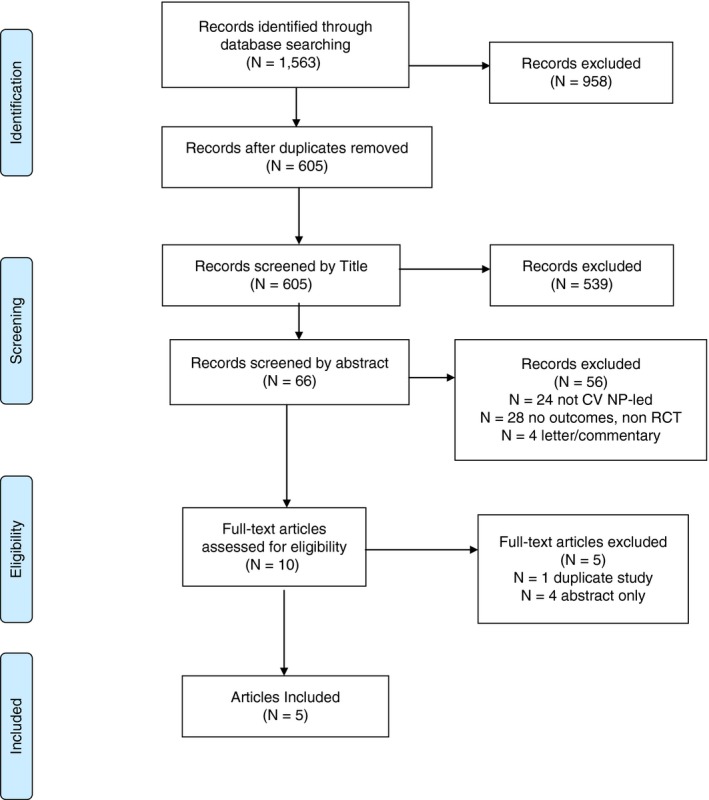
PRISMA article flow diagram [Colour figure can be viewed at http://www.wileyonlinelibrary.com]

All study references were uploaded to EndNote (X7.8‐ Clarivate Analytics). The initial database search identified 1563 studies. We identified 958 duplicate articles which were removed. After title review 539 studies were excluded and after abstract review a further 56 studies were removed because they did not meet the PICO criteria of the review. After full article review, five articles met the SR and meta‐analysis inclusion criteria. Studies were excluded for the following reasons: they were not RCT’s; we were unable to identify the NP role as the lead care provider; outcomes were not identifiable; was a letter or commentary; it was the same study, but different outcomes were reported in separate articles; the care was not CV NP‐led care and; abstracts that did not contain enough information to adequately determine the quality of the study (unable to find published article). The PRISMA flow diagram is presented in Figure 1.

### 2.5 Quality appraisal

**Figure 2:**
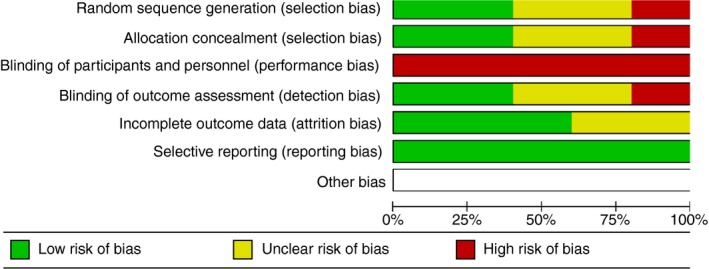
Risk of bias graph [Colour figure can be viewed at http://www.wileyonlinelibrary.com]

**Figure 3:**
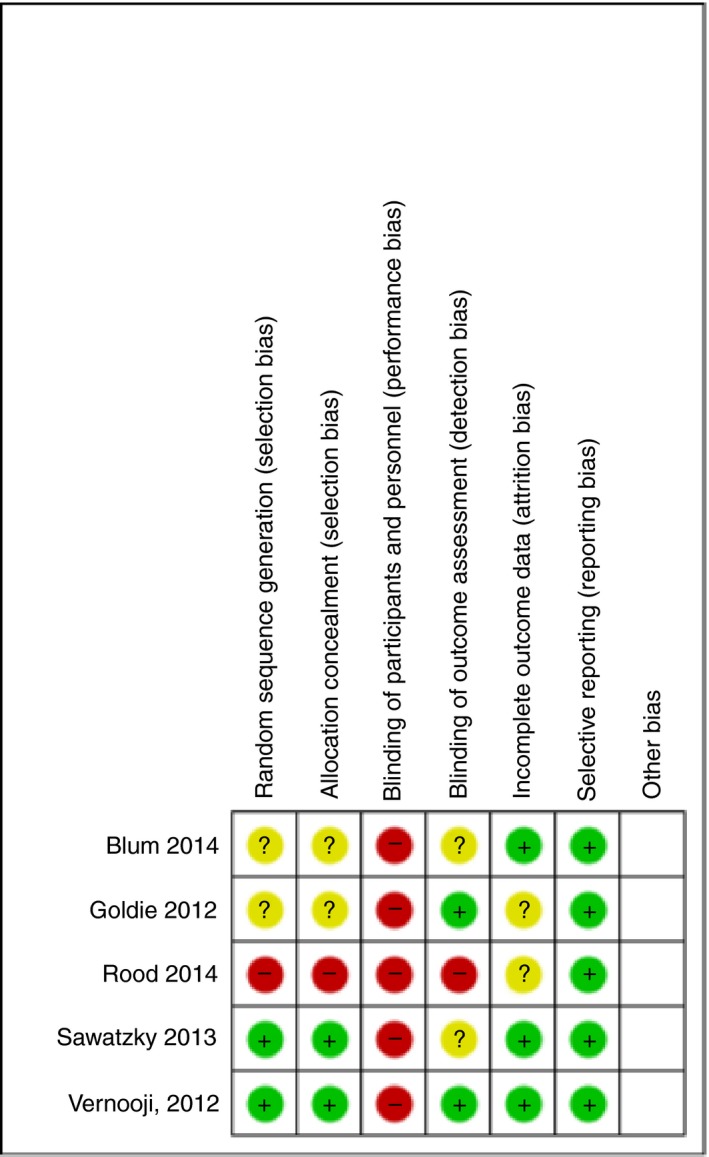
Risk of bias summary [Colour figure can be viewed at http://www.wileyonlinelibrary.com]

The Cochrane Modified Risk of Bias Tool (Part I & II) *Cochrane Handbook for Systematic Reviews of Interventions: Version 5.1.0* was used to determine risk of bias (Higgins et al., 2019) (Appendices S1B and S1C). Previous studies (Donald et al., 2014) have identified that it is not possible to blind participants and personnel to the ‘NP’ intervention, therefore the lack of blinding was not considered in the determination of risk of bias. Each study was assessed according to the type of bias and was rated as either unclear risk, low risk or high risk. Studies were then categorized into groups labelled as low risk of bias (at risk in zero to one categories), moderate risk of bias (at risk in two to three categories) or high risk of bias (four to six categories). One hundred percent of studies had no reporting bias, 60% had no attrition bias, 40% had no detection bias or selection bias. Overall, two studies were low risk of bias, two were moderate risk of bias and one was high risk of bias (Figures 2 and 3).

### 2.6 Data abstraction

Study data were extracted by two authors independently and disagreements were dealt with by consensus. Data extracted included: author, year, country, publication status, sample size, number of patients in each group, CV Care area, inclusion criteria, length of enrolment and follow‐up, study aims, NP role, associated outcomes of care and NP experience/training (if included) in each study. The data were documented on the data extraction form and ultimately compiled into Table 1. Identified outcomes included 30‐day readmission rate for heart failure (HF), length of stay after cardiac surgery, HRQOL SF‐36 physical and mental health scores and vascular risk reduction.

**Table 1:** Summary of data abstracted from included randomized controlled trials CV NP‐led care versus usual care

Author, year, country, additional publication	Sample size (*N*=) Number in each group (*n*=)	CV care area Number of sites	Inclusion criteria/Length of enrolment/Length of follow‐up	Study aims	Intervention (NP‐role)	Selected outcomes study findings for NP‐led care	Number of NPs experience and training
Blum & Gottlieb, 2014, USA	*N* = 206 NP *n* = 104 UC *n* = 102	Outpatient HF Multisite	Hospitalized in last year Four years Five years (or until death)	Reduce hospital and emergency room visits Improve self‐care.	Home monitoring Abnormal, weight and symptom changes treated Assessed by cardiologist as needed	No difference in 30‐day readmission rates for HF No difference SF 36 physical and mental composite score	One NP Extensive HF experience
Goldie et al., 2012, Canada	*N* = 103 NP *n* = 22 UC *n* = 81	Postoperative CV surgery One site	Scheduled for coronary bypass or valve surgery Nine months Followed admission to six–eight weeks post discharge.	Difference in: length of stay, readmit rates, complication, follow‐up, cardiac rehab, patient and team satisfaction	Followed clinical pathways Cardiac surgeon consulted as needed	Did not achieve sample size No difference in length of stay post cardiac surgery	One part time NP One year work experience in CV unit
Rood, 2014 USA Dissertation pilot project not published	*N* = 48 NP *n* = 20 UC *n* = 28	Inpatient HF transitioning to outpatient care One site	HF patients transitioning home three months/ 30 days	Reduce 30 day readmission rate for HF	Education and HF management follow‐up in three–five days post discharge Treated prn for 30 days Physician prn	No difference between 30‐day readmission for HF Flaws noted in trial design	One extensive HF experience Formal practice agreement.
Sawatzky et al., 2013, Canada	*N* = 200 NP *n* = 95 UC *n* = 105	Postoperative cardiac surgery One site	First coronary artery bypass surgery Must have phone 6 months From discharge until 6 weeks	Outcomes of adult cardiac surgery follow‐up model of care	Telephone follow‐up three days post discharge. Medical advice and/or education Patient seen prn to manage care Transitioned care to family physician by six weeks	No difference in length of stay after cardiac surgery No difference in SF 36 physical and mental composite scores	One cardiac surgery NP
Vernooij et al., 2012, Greving et al., 2015 The Netherlands	*N* = 330 NP *n* = 164 UC *n* = 166	Vascular risk reduction/ Two sites	Coronary, cerebral or peripheral artery atherosclerosis and at least 2 treatable risk factors not at target/ 17 months/ 1 year	Internet based, Outpatient vascular risk factor management program Promoted self‐management	On top of usual care. NP counselling via internet Followed Dutch cardiovascular risk management guidelines Supervised by internists	Blinded outcomes −12% adjusted Framingham risk score (−12% to −3%). Greater number of patients in NP‐led care reached LDL target (18%) and stopped smoking(19%)	Nine NPs

Abbreviations: CV, cardiovascular; HF, heart failure; LDL, low‐density lipoprotein cholesterol; NP, nurse practitioner‐led care; UC, usual care (Specific study: Blum–physician‐led, Goldie–hospitalist‐led, Rood‐retrospective chart review, Sawatzky‐physician‐led, and Vernooij,‐ physician‐led)

### 2.7 Synthesis

**Figure 4:**
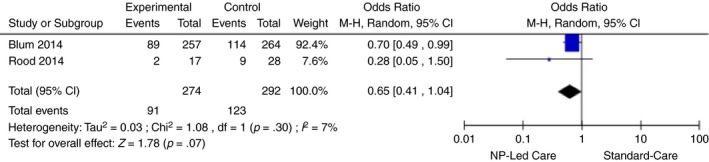
30‐day readmission for heart failure [Colour figure can be viewed at http://www.wileyonlinelibrary.com]

**Figure 5:**
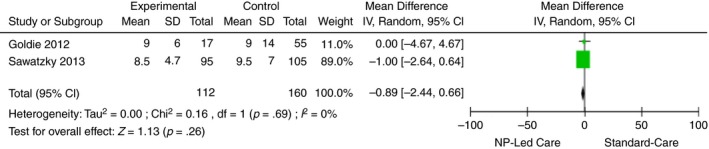
Length of stay post cardiac surgery [Colour figure can be viewed at http://www.wileyonlinelibrary.com]

**Figure 6:**
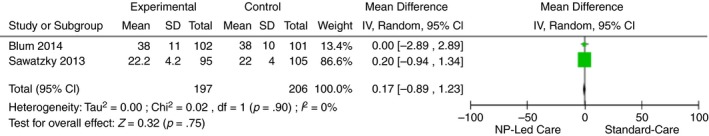
Health‐related quality of life: SF‐36 physical composite score [Colour figure can be viewed at http://www.wileyonlinelibrary.com]

**Figure 7:**
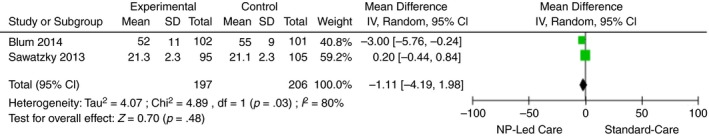
Health‐related quality of life: SF‐36 mental composite score [Colour figure can be viewed at http://www.wileyonlinelibrary.com]

We identified four associated outcomes of care but assessed SF 36 physical and mental composite scores as separate outcomes (total five outcomes). A separate meta‐analysis was completed for each outcome of care to pool results, except for vascular risk reduction because there was only one RCT. The effect sizes for length of stay after cardiac surgery and SF 36 physical and mental SF 36 scores were estimated as continuous outcomes with 95% confidence intervals, pooling mean difference and standardized mean differences. The  effect size for 30‐day readmission rates for HF (dichotomous) was estimated with 95% confidence interval, and pooling odds ratios. Heterogeneity was assessed using the I^2^ statistic. Heterogeneity was determined to be low if the I^2^ value was < 30 (Higgins et al., 2019). We carried out a meta‐analysis for NP‐led care compared with usual care with the identified associated outcomes. The effects of outcomes associated with NP‐led care were calculated using a random effects model to compute the mean difference or odds ratio (Table 2). Forest plots were produced for 30‐day readmission rate for HF, length of stay after cardiac surgery and HRQOL as SF 36 physical and mental composite scores (Figures 4, 5, 6, 7).

**Table 2:** Summary of findings

NP–led care compared with usual care in cardiovascular care						
Patient or population: cardiovascular care Setting: cardiovascular care Intervention: NP–led care Comparison: usual care						
Outcomes	Anticipated absolute effects^a^ (95% CI)		Relative effect (95% CI)	No. of participants (studies)	Certainty of the evidence (GRADE)	Comments
	Risk with usual care	Risk with NP–led care				
30‐day readmission rates for HF assessed with: number of patients admitted with HF within 30 days of discharge follow‐up: range 30 days to 5 years	421 per 1,000	**312 per 1,000** (198 to 493)	**RR 0.74** (0.47 to 1.17)	566 (2 RCTs)	⨁⨁◯◯ LOW^b^	There is no statistical difference between NP‐led care and Usual‐care in cardiovascular care.
Length of stay after cardiac surgery assessed with: number of days patient was admitted scale from: 6–8 follow‐up: mean 6 weeks	The mean length of stay after cardiac surgery was **9** days	The mean length of stay after cardiac surgery in the intervention group was 0.89 days lower (2.44 lower to 0.66 higher)	‐	272 (2 RCTs)	⨁⨁⨁◯ MODERATE^c^	There is no statistical difference between NP‐led care and usual‐care in cardiovascular care.
SF 36 physical composite score assessed with: patient rated values follow‐up: mean 2 years	The mean SF 36 physical composite score was **30** points	The mean SF 36 physical composite score in the intervention group was 0.17 points higher (0.89 lower to 1.23 higher)	‐	403 (2 RCTs)	⨁⨁⨁◯ MODERATE^d^	There is no statistical difference between NP‐led care and usual‐care in cardiovascular care.
SF 36 mental composite score assessed with: points follow‐up: 2 years	The mean SF 36 mental composite score was **38.5** points	The mean SF 36 mental composite score in the intervention group was 1.11 points lower (4.19 lower to 1.98 higher)	‐	403 (2 RCTs)	⨁⨁⨁◯ MODERATE^d^	There is no statistical difference between NP–led care and usual‐care in cardiovascular care.
Framingham risk score assessed with: percentage follow‐up: 1 years	Patients in the NP‐led group had a 12% decrease in risk (of developing coronary artery disease over 10 years. NP‐led group 18.4% of patients reached LDL targets and 19% stopped smoking			330 (1 RCT)	⨁⨁⨁◯ MODERATE^e^	NP‐led care had small effect on lowering cardiovascular risk and some vascular risk factors.
GRADE Working Group grades of evidence High certainty: We are very confident that the true effect lies close to that of the estimate of the effect. Moderate certainty: We are moderately confident in the effect estimate: The true effect is likely to be close to the estimate of the effect, but there is a possibility that it is substantially different. Low certainty: Our confidence in the effect estimate is limited: The true effect may be substantially different from the estimate of the effect. Very low certainty: We have very little confidence in the effect estimate: The true effect is likely to be substantially different from the estimate of effect.						

Abbreviations: CI, confidence interval; MD, mean difference; RR, risk ratio

a The risk in the intervention group (and its 95% confidence interval) is based on the assumed risk in the comparison group and the relative effect of the intervention (and its 95% CI).

b Lack of blinding with randomization and patient selection.

c Randomization process was unclear.

d Randomization issues, cannot control for confounders.

e Using change in Framingham Risk score as a measure of vascular risk.

All data were analysed using Review Manager software (RevMan, version 5.3; The Cochrane Collaboration) (Collaboration, 2014). We conducted a narrative synthesis of the vascular risk reduction outcomes as meta‐analysis was not possible. The Grading of Recommendations Assessment, Development and Evaluation (GRADE) system (Guyatt et al., 2011) was used to assess the quality and strength of the evidence and outcome presented in the ‘Summary of Findings’ (Table 2).

## 3 RESULTS

### 3.1 Study characteristics

There were five studies included in this review: two from Canada, two from the United States and one study from the Netherlands. Sample sizes varied from 48 to 330 total patients per study. Only one study did not have equal samples between groups. Cardiovascular care areas included outpatient HF care, postoperative heart surgery and one outpatient risk reduction clinic. Length of follow‐up varied between studies. One HF study followed patients for 30 days post discharge while the other study followed patients for five years. Follow‐up after discharge in the postoperative cardiac surgery studies was for 6–8 weeks. Patients in the risk factor reduction study were followed for one year. NP interventions included assessment of vital signs, pain management, assessment of signs of HF and adjustment of medications as required, patient teaching related to disease conditions and recovery, assessment of cardiovascular risk factors and treatment as indicated. Outcomes included 30‐day readmission for HF, length of stay in postoperative cardiac surgery and HRQOL scores!^2^. There was no significant difference between groups for these outcomes. The final outcome was a change in adjusted Framingham risk score of 12%. (decreased cardiovascular risk). One NP provided the NP‐intervention in five of the six studies. In the risk reduction setting there were nine NPs who performed the intervention. NPs experience level varied from one year to having extensive experience in their area of care.

### 3.2 Effect of interventions

We calculated differences in the effect of outcomes between NP‐led care and usual care. To clarify, we were interested in the change in HRQOL between NP‐led care and usual care not in comparing HRQOL outcomes between cardiac conditions. The details of the various outcomes, quality of evidence and magnitude of the effect are presented in Tables 2 and 3. The magnitude of effect for the outcomes was considered but was not strong enough to warrant upgrading any of the ratings.

**Table 3:** GRADE evidence profile: NP‐led care effectiveness in cardiovascular care

Quality assessment						Summary of findings					
						# Patients			Absolute risk		
Outcome & # of RCT	Risk of bias	Inconsistency	Indirectness	Imprecision	Publication bias	Usual care	NP‐led care	Relative risk(RR) Mean difference(MD) Relative change (RC) (95% CI)	Control risk^a^	Risk difference	Quality
30‐day readmission for HF 2	Very serious risk of bias: (lack of blinding with randomization and patient selection)	No serious inconsistency	No serious indirectness	No serious imprecision	Undetected	123/292 admission	91/274 admission	RR 0.74 (0.47–1.17)	42/100	Not significant	⊕⊕ΟΟ Low
Length of stay after cardiac surgery 2	No serious risk of bias	No serious inconsistency	No serious indirectness	Serious: imprecision (randomization process unclear)	Undetected	160	112	MD: −0.89 (−2.44 to 0.66)	9 to 9.5/100	Not significant	⊕⊕⊕Ο Moderate
SF36 physical composite 2	Serious risk of bias (randomization issues. cannot control for confounders)	No serious inconsistency	No serious indirectness	No serious imprecision	Undetected	206	197	MD: 0.17 (−0.89 to 1.23)	22 to 38/100	Not significant	⊕⊕⊕Ο Moderate
SF36 mental composite 2	Serious risk of bias (randomization issues, cannot control for confounders)	No serious inconsistency	No serious indirectness	No serious imprecision	Undetected	206	197	MD: 0.16 (−0.47 to−0.78)	21 to 50 /100	Not significant	⊕⊕⊕Ο Moderate
Change in Framingham risk score 1	No serious risk of bias	No serious inconsistency	Serious indirectness (using change of Framingham score as measure of vascular risk)	No serious imprecision	Undetected	159	155	RC −12% (−22%, 3%)^b^	13.2/100	Not significant	⊕⊕⊕Ο Moderate

Abbreviations: CI, confidence interval; GRADE, grading of recommendations assessment, development and evaluation; HF, heart failure; RCT, randomized controlled trials; RR, risk ratio

a The control rate is based on the median control group risk across studies.

b At baseline the Framingham risk score was higher in the NP‐led care group [16.1(*SD* 10.6) vs 14.0 (*SD* 10.5)]; therefore linnear regressiona analysis was used to adjust the outcomes for the separate variables of the Framingham risk score and for the baseline level of the Framingham risk score.

#### 3.2.1 Effect of NP‐led care on 30‐day readmission rates for HF

There were two RCTs involving 566 patients that assessed the effect of NP‐led care on 30‐day readmission rates for HF (Blum & Gottlieb, 2014; Rood, 2014). The meta‐analysis using a model of random effects revealed NP‐led care had no statistically difference (Risk Ratio: 0.74, 95% CI: 0.47, 1.17, Z = 1.27, *p* = .20) on 30‐day readmission rates in HF (Figure 4). I^2^ statistic is 15% and indicates that the risk of heterogeneity is low.

#### 3.2.2 Effect of NP‐led care on length of stay after cardiac surgery

Length of stay was assessed in two NP‐led postoperative CV surgical RCTs (272 patients) (Goldie, Prodan‐Bhalla, & Mackay, 2012; Sawatzky, Christie, & Singal, 2013). The mean difference for length of stay indicates no significant difference between NP‐led care and usual care on length of stay in postoperative cardiac surgery (mean difference [MD] = −0.89, 95% CI: −2.44, 0.66, Z = 1.13, *p* = .26,) I^2 ^statistic is 0%, therefore low risk of heterogeneity (Figure 5).

#### 3.2.3 Effect of NP‐led care on SF 36 physical composite score

Two studies with 403 patients investigated the effectiveness of NP‐led care on HRQOL (Blum & Gottlieb, 2014; Sawatzky et al., 2013). The mean difference ([MD] = 0.17, 95% CI: −0.89, 1.23; Z = 0.32), *p* = .75, (Figure 5), inference no significant difference. I^2^ = 0% indicating low risk for heterogeneity (Figure 6).

#### 3.2.4 Effect of NP‐led care on SF 36 mental composite score

The two similar RCTs evaluating SF 36 Physical composite Score (403 patients) also evaluated SF 36 mental composite score (Blum & Gottlieb, 2014; Sawatzky et al., 2013). The mean difference for SF 36 mental composite score (mean difference [MD] = −1.11, 95% CI: −4.19, 1.98; Z = 0.70, *p* = .48 (Figure 6); suggests no statistical difference. I^2^ Statistic is 80%, therefore, there is a high risk of heterogeneity and these results must be interpreted with care because there is considerable variation in the combined or pooled results and it may be misleading to report a combined summary measure (Figure 7).

#### 3.2.5 Effect of NP‐led care on vascular risk reduction

The Vernooij study compared NP–led care to usual care to reduce vascular risk factors in patients with clinically manifested vascular disease (330 patients) (Greving et al., 2015; Vernooij et al., 2012). As there is only one study, a narrative synthesis has been completed.

A relative change in the Framingham risk score from baseline to one year follow‐up was assessed as the primary outcome. The NP–led group had a higher Framingham risk score at baseline. Therefore, the baseline Framingham risk score was adjusted, to produce a relative change of −12% (−22% to −3%) versus usual care −8% (−18% to 2%). That is, patients in the NP‐led group had a 12% decrease in risk of developing coronary heart disease over the next 10 years. Secondary endpoints were absolute changes in the levels of risk factors. In the NP‐led group 18.4% of patients reached low‐density lipoprotein cholesterol (LDL) targets and 19% stopped smoking.

### 3.3 Publication bias

A limited number of trials were assessed in this meta‐analysis, thus we were unable to assess the potential for publication bias.

### 3.4 Psychometrics

The SF 36 assessed the HRQOL scores. This instrument is used extensively, is well‐validated, reliable and responsive (Reynolds, Ellis, & Zimetbaum, 2008). The Framingham risk score is a well‐known risk stratification tool that has been shown to be effective in predicting the 10‐year risk for coronary artery disease and guiding when to initiate treatment (Gunaydin et al., 2016).

### 3.5 Quality of the evidence

There is low quality of evidence (due to risk of bias) for no statistical difference in 30‐day readmission for HF rates associated with NP‐led care. The quality of the evidence for length of stay is moderate because one study has a very wide confidence interval. Overall the quality of the evidence for the SF36 physical composite quality of life scores is moderate because of the rating for risk of bias. The SF 36 mental composite score was also found to have moderate risk of bias as the quality of evidence rating was moderate. The quality of the risk reduction study is moderate, mostly due to the rating for indirectness as they are using a change in Framingham scores to infer vascular risk reduction. The results of the intervention could also be affected by the participants’ ability to use the website (Table 3).

## 4 DISCUSSION

### 4.1 Summary of main findings

With society's ageing population and strained healthcare systems, using NP‐led care can deliver high‐quality care to meet CV patient healthcare needs. However, in this era of constrained healthcare resources, decisions‐makers need solid evidence to implement new models of care. To this end, we conducted a SR of the outcomes of NP–led CV‐care. We identified five RCTs that evaluated a total of 1,268 patients across three areas of CV care including heart failure, postoperative CV surgery and vascular risk reduction. We identified two patient reported HRQOL outcomes (SF 36 physical and mental composite scores), two systems outcomes of length of stay after cardiac surgery and 30‐day readmission rates in HF and one vascular risk reduction outcome. We conducted four meta‐analyses to analyse the two HRQOL and two systems outcomes related to NP‐led CV care and reported a narrative synthesis of the vascular risk reduction outcomes as there was only one study. However, we found no statistical difference in NP‐led care and usual care for any of the outcomes.

Nurse practitioner‐led care is well‐known to be associated with positive outcomes of care (College of Registered Nurses of Nova Scotia, 2016). Decreasing 30‐day readmission rates for HF and length of stay after cardiac surgery have been identified as priority healthcare reform issues since the early 1970s (Canadian Institute for Health Information, 2012). Nurse practitioner roles have been implemented to assist with achieving these health system goals.

Previously, studies have shown that CV NP–led care is associated with decreasing 30‐day readmission rates for HF (David, Britting, & Dalton, 2015; Echeverry, Lamb, & Miller, 2015; Estrella‐Holder & Zieroth, 2015) which does not correlate with our findings. We focused on RCTs while the studies that identified reduction in 30‐day readmission rates were of various other methodologies (retrospective, improvement project and descriptive). The studies also followed patients for different lengths of time. Blum (Blum & Gottlieb, 2014) initially found decreased 30‐day HF readmission rates however it was not maintained after one year while Estrela‐Holder and Zeroth followed patients for 6 months (Estrella‐Holder & Zieroth, 2015). Rood also found a 30‐day readmission rate reduction, however patients were followed for 30 days and flaws noted in the research design could contribute to the findings (Rood, 2014).

Our study findings did not identify whether NP‐led care was associated with decreasing length of stay after cardiac surgery. However, Meyer and Miers (Meyers & Miers, 2005) assessed decreasing length of stay in postoperative CV surgery patients with a retrospective chart review. They compared the previous model of care to current NP–led care. A decrease of 1.91 days in length of stay was found. The studies in our meta‐analysis had some differences from the the Meyer and Miers study which may have influenced the different findings. The Goldie study (Goldie et al., 2012) was not able to recruit to the full sample size while the Sawatzky study, that did not include length of stay as one of the original main outcomes of care (Sawatzky et al., 2013). The difference in findings may therefore be because the sample sizes were not adquate to identify a length in stay. 

When HRQOL is evaluated as an outcome associated with direct NP‐led care it has been found to be associated with higher levels of HRQOL scores. However, when HRQOL is assessed as an outcome when NP‐led care is compared with another healthcare provider, frequently no difference in the patient's self‐reported health status has been found (Dierick‐van Daelle, Metsemakers, Derckx, Spreeuwenberg, & Vrijhoef, 2009; Lenz, Mundinger, Kane, Hopkins, & Lin, 2004; Newhouse et al., 2011; Sangster‐Gormley et al., 2015; Sidani & Doran, 2010). Our review showed no difference in SF 36 physical and mental composite scores associated with CV NP‐led care when compared with other healthcare providers which is congruent with other research findings.

One study identified that NP‐led care assists patients to decrease their overall vascular risk by lowering certain vascular risk factors (Vernooij et al., 2012). This correlates with other research findings where patients in the NP–led group had better control of their cholesterol levels and other risk factors (Martinez‐Gonzales, Rosemann, Tandjung, & Djalali, 2015; Nieuwkerk et al., 2012; Stanik‐Hutt et al., 2013; Swan et al., 2015).

Several pertinent abstracts of NP‐led care were not published. A SR of why medical and health‐related studies are not being published concluded that the most common reasons were lack of time or rated as a low priority (Song, Loke, & Hooper, 2014). Other researchers have noted that NPs find it difficult to balance their clinical role with conducting research (Kilpatrick et al., 2010). Additional employer support or academic mentorship could help NPs balance the demands of both roles and enable them to publish important research that is needed to guide practice.

While incorporating NPs into CV care has been welcome, little is known about the outcomes of care. Our review of the available evidence shows there is no significant impact (either positive or negative) on patient outcomes. While the NP role is the most studied healthcare role (College of Registered Nurses of Nova Scotia, 2016), there are many low quality studies and few RCTs (Donald et al., 2014; Worster, Sardo, Thrasher, Fernandes, & Chemeris, 2005). Our findings also support this and highlight the important need for more investment in high‐quality research in this important model of healthcare delivery.

### 4.2 Strengths & limitations

The strengths of this SR include using a comprehensive search strategy, rigorously screening and adhering to the PRISMA checklist, using established quality assessment tools and completing the outcomes assessment with GRADE (Guyatt et al., 2011; Moher et al., 2015). The limitation of this study is that very few studies met the inclusion criteria for this systematic review. The studies that were included were of low to moderate quality mostly due to serious risk of bias, randomization issues and serious indirectness (Table 3).

The findings in our review are inconclusive due to the limited number of RCTs in NP‐led CV care and the design flaws in the reviewed studies. Randomized controlled trials provide the strongest level of evidence (Burns, Rohrich, & Chung, 2011) but, a poorly designed RCT can give misleading results (Centre For Evidenced‐Based Medicine, 2016). Randomized controlled trials are more difficult and expensive to conduct, which may curb the use of this design. Limited numbers of RCTs is not unique to nursing. For example, RCTs account for only five percent of completed studies in medicine (Bondemark & Ruf, 2015; Kovesdy & Kalantar‐Zadeh, 2012).

The risk of bias assessment in this SR found many randomization issues in the included studies resulting in an elevated risk of bias rating. There were also other design flaws in the articles that we reviewed which may have led to our non‐statistical findings. When publishing, a very detailed methods section is required to allow for replication and to allow the reader to determine if it is pertinent to their patient population (Centre For Evidenced‐Based Medicine, 2016). Our study findings showed that intervention details were not always documented and ultimately affected our rating of risk of bias and quality assessment.

### 4.3 Recommendations for future studies

All the studies examined were designed and conducted prior to 2013 when SPIRIT was launched as a protocol to help improve the quality of clinical trial protocols. The CONSORT Statement (2010) is a 25‐item checklist and flow diagram for authors to use to ensure transparent reporting of randomized trials (Schulz, Altman, Moher, & Group, 2010). Using the SPIRIT and CONSORT (Chan, Tetzlaff, Altman, et al., 2013; Chan, Tetzlaff, Gotzsche, et al., 2013) protocols and checklists when designing and reporting a RCT will help to ensure that all important elements of the trial are reported and thus decrease the risk of bias which ultimately will help improve the overall quality of NP‐led RCTs. We recommend that well‐designed, high‐quality RCTs need to be completed in CV NP‐led care. Nurse practitioners need to ensure completed research be published to establish and document outcomes associated with CV NP‐led care. Published evidence should be used to drive clinical practice (Centre For Evidenced‐Based Medicine, 2016).

## 5 CONCLUSION

The CV NP role has been increasingly used; however, research related to the role is lagging behind clinical practice. It is extremely important for further high‐quality research to be conducted to identify clinical outcomes of care associated with NP‐led CV care as a model of care. Cardiovascular NPs therefore need to be supported to conduct and publish high‐quality clinical research which will provide decision‐makers with essential evidence for implementing CV NP roles.

## CONFLICT OF INTEREST

No conflict of interest has been declared by the authors.

## AUTHOR CONTRIBUTIONS

MJS, MS, MSM, RTT and CMN made substantial contributions to the conception and design, acquisition of data, analysis and interpretation of data; MJS, MS, MSM, RTT and CMN were involved in drafting the manuscript and revising it critically for important intellectual content; MJS, MS, MSM, RTT and CMN gave final approval of the version to be published. Each author participated sufficiently in the work to take public responsibility for appropriate portions of the content; MJS, MS, MSM, RTT and CMN agreed to be accountable for all aspects of the work in ensuring that questions related to the accuracy or integrity of any part of the work are appropriately investigated and resolved.
