# Supplementary material for: Outcomes of nurse practitioner‐led care in patients with cardiovascular disease: A systematic review and meta‐analysis
Source: J Adv Nurs. 2019 Oct 24;76(1):81–95. doi: 10.1111/jan.14229 (PMC6973236; doi:10.1111/jan.14229)
Supplement: Supplementary file 1 [file JAN-76-81-s001.docx]

# Appendix A

## Electronic database search strategies for Outcomes of Care for CV NP-led Systematic Review

**CINAHL with full text**
( (MH "Heart Diseases+") or cardio* or cardia* or heart* or coronary or angina* or ventric* or myocard* or pericard* or ischem* or ischaem* or emboli* or thrombo* or "atrial fibrillat*" or tachycard* or arrhythmi* or endocard* or "sick sinus" or hypertensi* or "peripheral artery disease*" or ((high or increased or elevated) w2 "blood pressure") or hyperlipid* or hyperlipemi* or hyperlipaemi* or hypercholester* or hyperlipoprotein* or hypertriglycerid* or cholesterol or "blood pressure" ) AND ( [...](javascript:showHistoryTerm('ctl00_ctl00_MainContentArea_MainContentArea_historyControl_HistoryRepeater_ctl02_ellipsis',true))

Limit to RCT,, 2007-2017

**Web of Science** (cardio* or cardia* or heart* or coronary or angina* or ventric* or myocard* or pericard* or ischem* or ischaem* or emboli* or thrombo* or "atrial fibrillat*" or tachycard* or arrhythmi* or endocard* or "sick sinus" or hypertensi* or "peripheral artery disease*" or r hyperlipid* or hyperlipemi* or hyperlipaemi* or hypercholester* or hyperlipoprotein* or hypertriglycerid* or cholesterol or "blood pressure ") *AND* **TOPIC:** ("nurse practitioner*") *AND***TOPIC:** (random* or trial or groups or "quasi experimental") 
Refined By: **PUBLICATION YEARS:** (2015 OR 2013 OR 2016 OR 2008 OR 2011 OR 2009 OR 2007 OR 2014 OR 2010 OR 2012 OR 2017)

**Scopus** TITLE-ABS-KEY ( cardio*  OR  cardia*  OR  heart*  OR  coronary  OR  angina*  OR  ventric*  OR  myocard*  OR  pericard*  OR  ischem*  OR  ischaem*  OR  emboli*  OR  thrombo*  OR  "atrial fibrillat*"  OR  tachycard*  OR  arrhythmi*  OR  endocard*  OR  "sick sinus"  OR  hypertensi*  OR  "peripheral artery disease*"  OR  hyperlipid*  OR  hyperlipemi*  OR  hyperlipaemi*  OR  hypercholester*  OR  hyperlipoprotein*  OR  hypertriglycerid*  OR  cholesterol  OR  "blood pressure" )  AND  TITLE-ABS-KEY ( "nurse practitioner*" )  AND  TITLE-ABS-KEY ( random*  OR  trial  OR  groups )  AND  ( LIMIT-TO ( PUBYEAR ,  2017 )  OR  LIMIT-TO ( PUBYEAR ,  2016 )  OR  LIMIT-TO ( PUBYEAR ,  2015 )  OR  LIMIT-TO ( PUBYEAR ,  2014 )  OR  LIMIT-TO ( PUBYEAR ,  2013 )  OR  LIMIT-TO ( PUBYEAR ,  2012 )  OR  LIMIT-TO ( PUBYEAR ,  2011 )  OR  LIMIT-TO ( PUBYEAR ,  2010 )  OR  LIMIT-TO ( PUBYEAR ,  2009 )  OR  LIMIT-TO ( PUBYEAR ,  2008 )  OR  LIMIT-TO ( PUBYEAR ,  2007 ) )

**Ovid MEDLINE**

1. exp heart defects, congenital/ or exp heart diseases/

2. (cardio* or cardia* or heart* or coronary or angina* or ventric* or myocard* or pericard* or isch?em* or emboli* or thrombo* or atrial fibrillat* or tachycard* or arrhythmi* or endocard* or sick sinus or hypertensi* or peripheral artery disease* or ((high or increased or elevated) adj2 blood pressure) or hyperlipid* or hyperlip?emi* or hypercholester* or hyperlipoprotein* or hypertriglycerid* or cholesterol or blood pressure).ti,ab,kf.

3. 1 or 2

4. Nurse practitioners/ or nurse practitioner*.ti,ab,kf.

5. 3 and 4

6. limit 5 to yr="2007 -Current"

7. randomized controlled trial.pt.

8. clinical trial.pt.

9. (randomi?ed or quasi experimental).ti,ab,kf.

10. placebo.ti,ab,kf.

11. dt.fs.

12. randomly.ti,ab,kf.

13. trial.ti,ab,kf.

14. groups.ti,ab,kf.

15. or/7-14

16. animals/

17. humans/

18. 16 not (16 and 17)

19. 15 not 18

20. 6 and 19

**Ovid EMBASE**

1. exp heart disease/

2. (cardio* or cardia* or heart* or coronary or angina* or ventric* or myocard* or pericard* or isch?em* or emboli* or thrombo* or atrial fibrillat* or tachycard* or arrhythmi* or endocard* or sick sinus or hypertensi* or peripheral artery disease* or ((high or increased or elevated) adj2 blood pressure) or hyperlipid* or hyperlip?emi* or hypercholester* or hyperlipoprotein* or hypertriglycerid* or cholesterol or blood pressure).ti,ab,kw.

3. 1 or 2

4. Nurse practitioners/ or nurse practitioner*.ti,ab,kw.

5. 3 and 4

6. exp clinical trial/

7. (randomi?ed or quasi experimental).ti,ab,kw.

8. placebo.ti,ab,kw.

9. dt.fs.

10. randomly.ti,ab,kw.

11. trial.ti,ab,kw.

12. groups.ti,ab,kw.

13. or/6-12

14. animal/

15. human/

16. 14 not (14 and 15)

17. 13 not 16

18. 5 and 17

19. limit 5 to randomized controlled trial

20. 18 or 19

21. limit 20 to yr="2007 –Current

**Cochrane Library Database of Systematic Review and Controlled Trials (Ovid Central)**

1. exp Cardiovascular Diseases/

2. (cardio* or cardia* or heart* or coronary or angina* or ventric* or myocard* or pericard* or isch?em* or emboli* or thrombo* or atrial fibrillat* or tachycard* or arrhythmi* or endocard* or sick sinus or hypertensi* or peripheral artery disease* or ((high or increased or elevated) adj2 blood pressure) or hyperlipid* or hyperlip?emi* or hypercholester* or hyperlipoprotein* or hypertriglycerid* or cholesterol or blood pressure or stroke* or stokes or cerebrovasc* or cerebral vascular or apoplexy or ((brain or cerebral or lacunar) adj2 (accident* or infarct*))).ti,ab,kw.

3. 1 or 2

4. Nurse practitioners/ or nurse practitioner*.ti,ab,kw.

5. 3 and 4

6. (Adolescent/ or exp Infant/ or exp Child/) not ((Adolescent/ or exp Infant/ or exp Child/) and (exp Adult/ or exp Aged/))

7. 5 not 6

8. limit 7 to yr="2007 –Current

**ProQuest Dissertations & Thesis Global**

all(cardio* OR cardia* OR heart* OR coronary OR angina* OR ventric* OR myocard* OR pericard* OR ischem* OR ischaem* OR emboli* OR thrombo* OR “atrial fibrillat*” OR tachycard* OR arrhythmi* OR endocard* OR “sick sinus” OR hypertensi* OR “peripheral artery disease*” OR hyperlipid* OR hyperlipemi* OR hyperlipaemi* OR hypercholester* OR hyperlipoprotein* OR hypertriglycerid* OR cholesterol OR blood pressure”)AND all (“nurse practitioner*”) AND all(pd(20070101-20171231))
