# Supplementary material for: Outcomes of nurse practitioner‐led care in patients with cardiovascular disease: A systematic review and meta‐analysis
Source: J Adv Nurs. 2019 Oct 24;76(1):81–95. doi: 10.1111/jan.14229 (PMC6973236; doi:10.1111/jan.14229)
Supplement: Supplementary file 2 [file JAN-76-81-s002.pdf]

Appendix B

Cochrane Collaboration modified tool for assessing risk of bias for RCTs, PART I

Use this form to assess risk of bias for randomized controlled trials.

Bias is assessed as a judgment (high, low, or unclear) for individual elements from five domains of bias (selection, performance, attrition, reporting, and other).

Risk of selection, reporting, and other bias are assessed in the **Quality Assessment Form Part I.** Risk of performance, detection, and attrition bias are assessed using the **Quality Assessment Form Part II.**

Using the guidance provided at the end of this form, select either "high", "low" or "unclear" for each judgment. When complete, proceed to **Part II of the Quality Assessment Form**

**REF ID:**					
Domain	***Description***	***High risk of bias***	***Low risk of bias***	***Unclear risk of bias***	***Reviewer Assessment***
Selection bias<br><br>***Random sequence generation***	Described the method used to generate the allocation sequence in sufficient detail to allow an assessment of whether it should produce comparable groups.<br><br>**Reviewer Comments:**	Selection bias (biased allocation to interventions) due to inadequate generation of a randomized sequence.	Random sequence generation method should produce comparable groups	Not described in sufficient detail	**Judgment: Random sequence generation**<br><br>☐ **High**<br>☐ **Low**<br>☐ **Unclear**
Selection bias<br><br>***Allocation concealment***	Described the method used to conceal the allocation sequence in sufficient detail to determine whether intervention allocations could have been foreseen in advance of, or during, enrollment.<br><br>**Reviewer Comments:**	Selection bias (biased allocation to interventions) due to inadequate concealment of allocations prior to assignment.	Intervention allocations likely could not have been foreseen in advance of, or during, enrollment	Not described in sufficient detail	**Judgment: Allocation concealment**<br><br>☐ **High**<br>☐ **Low**<br>☐ **Unclear**
Domain	***Description***	***High risk of bias***	***Low risk of bias***	***Unclear risk of bias***	***Reviewer Assessment***
Reporting Bias	State how the possibility of selective	Reporting bias due to	Selective outcome	Insufficient information to	**Judgment: Selective**

Selective reporting	outcome reporting was examined by the authors and what was found.<br><br>**Reviewer Comments:**	selective outcome reporting.	reporting bias not detected	permit judgment of 'Low risk' or 'High risk'.<br><br>(*It is likely that the majority of studies will fall into this category.)*	**reporting**<br><br>□ **High**<br>□ **Low**<br>□ **Unclear**
Other bias<br><br>***Other sources of bias***	Any important concerns about bias not addressed above. If particular questions/entries were pre-specified in the study's protocol, responses should be provided for each question/entry.<br><br>**Reviewer Comments:**	Bias due to problems not covered elsewhere in the table.	No other bias detected	There may be a risk of bias, but there is either:<br>Insufficient information to assess whether an important risk of bias exists; or<br>Insufficient rationale or evidence that an identified problem will introduce bias.	**Judgment: Other sources of bias**<br><br>□ **High**<br>□ **Low**<br>□ **Unclear**
