# Supplementary material for: Outcomes of nurse practitioner‐led care in patients with cardiovascular disease: A systematic review and meta‐analysis
Source: J Adv Nurs. 2019 Oct 24;76(1):81–95. doi: 10.1111/jan.14229 (PMC6973236; doi:10.1111/jan.14229)
Supplement: Supplementary file 3 [file JAN-76-81-s003.pdf]

Appendix C

Cochrane Collaboration modified tool for assessing risk of bias for RCTs, PART II

Use this form to assess risk of bias for randomized controlled trials.

Bias is assessed as a judgment (high, low, or unclear) for individual elements from five domains of bias (selection, performance, attrition, reporting, and other).

Risk of selection, reporting, and other bias are assessed in the **Quality Assessment Form Part I.** Risk of performance, detection, and attrition bias are assessed using the **Quality Assessment Form Part II.**

Using the guidance provided at the end of this form, select either "high", "low" or "unclear" for each judgment.

Risk of bias for the domains in the Form Part II will be assessed for each main or class of outcomes. Please indicate the specific outcome and complete the assessment for each.

REF ID:

Outcome(s):

Domain	***Description***	***High risk of bias***	***Low risk of bias***	***Unclear risk of bias***	***Reviewer Assessment***
Performance bias<br><br>***Blinding (participants and personnel)***	Described all measures used, if any, to blind study participants and personnel from knowledge of which intervention a participant received. Provided any information relating to whether the intended blinding was effective.<br><br>**Reviewer Comments:**	Performance bias due to knowledge of the allocated interventions by participants and personnel during the study.	Blinding was likely effective.	Not described in sufficient detail	**Judgment: Blinding (participants and personnel)**<br><br>☐ **High**<br>☐ **Low**<br>☐ **Unclear**
Detection bias<br><br>***Blinding (outcome assessment)***	Described all measures used, if any, to blind outcome assessors from knowledge of which intervention a participant received. Provided any information relating to whether the intended blinding was effective.<br><br>**Reviewer Comments:**	Detection bias due to knowledge of the allocated interventions by outcome assessors.	Blinding was likely effective.	Not described in sufficient detail	**Judgment: Blinding (outcome assessment)**<br><br>☐ **High**<br>☐ **Low**<br>☐ **Unclear**

Domain	***Description***	***High risk of bias***	***Low risk of bias***	***Unclear risk of bias***	***Reviewer Assessment***
Attrition bias<br><br>***Incomplete outcome data***	Described the completeness of outcome data for each main outcome, including attrition and exclusions from the analysis. Stated whether attrition and exclusions were reported, the numbers in each intervention group (compared with total randomized participants), reasons for attrition/exclusions where reported.<br><br>**Reviewer Comments:**	Attrition bias due to amount, nature or handling of incomplete outcome data.	Handling of incomplete outcome data was complete and unlikely to have produced bias	Insufficient reporting of attrition/exclusions to permit judgment of 'Low risk' or 'High risk' (e.g. number randomized not stated, no reasons for missing data provided)	**Judgment: Incomplete outcome data**<br><br>□ **High**<br>□ **Low**<br>□ **Unclear**
